# *Protium javanicum* Burm. Methanol Extract Attenuates LPS-Induced Inflammatory Activities in Macrophage-Like RAW264.7 Cells

**DOI:** 10.1155/2019/2910278

**Published:** 2019-04-21

**Authors:** Akash Ahujaa, Mi-Yeon Kim, Jae Youl Cho

**Affiliations:** ^1^Department of Integrative Biotechnology, Sungkyunkwan University, Suwon 16419, Republic of Korea; ^2^School of Systems Biomedical Science, Soongsil University, Seoul 06978, Republic of Korea

## Abstract

*Protium javanicum *Burm. f. is a medicinal plant used in traditional medicine. Gum and oleoresins from this plant have been used as anti-inflammatory agents for treating ulcers, headaches, eyelid inflammation, and rheumatic pain. However, its anti-inflammatory mechanism of action is still unknown. To better understand the mechanism, we used lipopolysaccharide- (LPS-) treated RAW264.7 cells to measure inflammatory mediators with the Griess assay and to identify target signaling molecules by immunoblot analysis. In this study, we report that the* Protium javanicum *methanol extract (Pj-ME) plays an important role in suppressing nitric oxide (NO) levels without cytotoxicity. The effect of Pj-ME in LPS-induced expression leads to reduced inflammatory cytokine expression, specifically inducible nitric oxide synthase (iNOS), cyclooxygenase (COX-2), and tumor necrosis factor (TNF-*α*). Pj-ME significantly inhibited LPS-induced protein expression of the nuclear factor-kappa B (NF-*κ*B) signaling pathway in a time-dependent manner. Syk and Src were identified as putative signaling molecules of Pj-ME-mediated anti-inflammatory activity, which were inhibited by Pj-ME. We demonstrated that Pj-ME controls the STAT3 signaling pathway by suppressing STAT3 and JAK phosphorylation and also downregulates the gene expression of IL-6. Therefore, these results elucidate Pj-ME as a novel anti-inflammatory naturally derived drug with anti-inflammatory and antioxidant properties which may be subject to therapeutic and prognostic relevance.

## 1. Introduction

Inflammation is a complex defense mechanism that neutralizes and restores cell or tissue to its normal function [1]. However, proinflammatory stimuli and stress conditions lead to pathogenesis and chronic disease [2]. In this regard, macrophages play an important role in mediating inflammation and secreting inflammatory cytokines after activation [3, 4]. Macrophages are activated in several ways, such as by bacteria and hepatitis B virus, and leads to the release of inflammatory cytokines. Moreover, lipopolysaccharide (LPS), an important component of the outer wall of Gram-negative bacteria, is also known to activate macrophages and lead to the release of typical proinflammatory cytokines, such as tumor necrosis factor (TNF-*α*) and interleukin 6 (IL-6), promoting tissue damage and chronic disease [5]. We used LPS-stimulated macrophages to study the classical inflammation model* in vitro*. In fact, although inflammation is considered as an important response for the host defense against infections, it also could become cause to many chronic diseases. Sustainable levels of tissue injury, oxidative stress, angiogenesis, and fibrosis as the results of a series of inflammatory responses are also know to lead to other deadly complications in some tissues and organs such as cancer, Alzheimer's disease, diabetes, and atherosclerosis [6, 7]. Therefore, development of new and safer treatment strategies to prevent and treat these inflammatory diseases could be essential.

NF-*κ*B is family of transcription factors, which share the Rel homology domain and are sequestered in the cytoplasm by I*κ*B (inhibitor of *κ*B) family members [8]. Proinflammatory cytokines, such as TNF-*α*, IL-1, and IL-6, and triggers of toll-like receptors (TLRs), such as lipopolysaccharide, are known to activate NF-*κ*B [9]. Signals from external stimuli are translocated through adaptor molecules, which activate the I*κ*B kinase (IKK) complex and phosphorylates cytosolic I*κ*B, followed by ubiquitination and sequential activation NF-*κ*B into the nucleus, and culminating in proinflammatory cytokine activation (for example, iNOS/NO, COX-2, and TNF-*α*) [10]. Signal transducer and activator of transcription (STAT) is a family of cytoplasmic proteins that regulate an array of genes in response to cytokines and growth factors [11]. In contrast, STAT3 activation generates a number of inflammatory responses and regulates a variety of signaling pathways [12]. The precise mechanism that STAT3 induces the inflammatory response has not been established. However, Src tyrosine kinase or mutations in JAK proteins can lead to hyperphosphorylation of STAT3 [13]. Identification of compounds or natural products that inhibit STAT3 activation have great potential for treating inflammatory diseases [14].

*Protium javanicum *Burm. f., which belongs to the family Burseraceae from Indonesia, is locally known in Indonesia as “kayu bawang” or “kayu pahit”. The plant has been used for making desks, tables, and exterior walls because of its durability. Traditionally, gum, and oleoresins from* P. javanicum *had been used in folk medicine as anti-inflammatory agents for treating ulcers and for headaches, eyelid inflammation, and rheumatic pain [15]. Recent studies using phytochemical fractionation of* P. javanicum *extracts had led to the identification of scopoletin, quercetin, and stigmasterol [16]. Plant formulated natural products are gaining wide consideration in developing inflammatory and chemopreventive remedies because of their little or no side effects. Moreover, there is much considerable motivation to investigate plant-based phytochemicals and their potential to reduce inflammatory symptoms or inhibit tumor progression and metastasis [17–19]. Despite the use of* Protium javanicum *in traditional medicine, there have been no reports on its anti-inflammatory mechanism of action. Therefore, our objective in this study was to determine the anti-inflammatory mechanism of* Protium javanicum *Burm. f. methanol extract in RAW264.7 macrophages.

In the present study, we have shown that the anti-inflammatory effect of Pj-ME is related to NF-*κ*B and STAT3 in LPS-stimulated macrophages. To better understand the anti-inflammatory mechanism involving LPS-stimulated macrophages, we used RT-PCR to analyze the cytokines downregulated in LPS-stimulated macrophages after treatment with Pj-ME. We also evaluated the anti-inflammatory effect on the NF-*κ*B signaling pathway using a luciferase reporter gene and protein expression to determine the specific molecular target. Treatment with Pj-ME reduced p85, IKK*α*/*β*, I*κ*B*α*, p50, and p65 proteins in the NF-*κ*B inflammatory pathway in a time-dependent manner. However, Syk and Src overexpression in HEK293 cells treated with Pj-ME abrogated Syk and Src phosphorylation and prevented inflammatory activity. Thus, Pj-ME targets Syk and Src to mediate its anti-inflammatory effect. We also report that Pj-ME inhibits STAT3 activation and gene expression of IL-6 in a time-dependent manner. Therefore, our results may provide evidence for the underlying mechanism of Pj-ME in activated macrophages.

## 2. Materials and Methods

### 2.1. Materials

RAW264.7 cells from mice (BLAB/c, ATCC number TIB-71) were purchased from ATCC (Rockville, MD, USA). Dimethyl Sulfoxide (DMSO), L-N^G^–nitroarginine methyl ester (L-NAME), lipopolysaccharide (LPS,* Escherichia coli* 0111:B4), and (3-4,5-dimethylthiazol-2-yl)-2,5-diphenyltetrazolium bromide (MTT) were purchased from Sigma Chemical Co. (St Louis, MO, USA). Gene specific PCR primers for iNOS, TNF-*α*, COX-2, and GAPDH were synthesized from Bioneer Inc. (Daejeon, Republic of Korea). Antibodies to phosphorylated and total protein (p65, p50, I*κ*B*α*, IKK*α*/*β*, Syc, Syk, STAT3, JAK, and *β*-actin) were obtained from Cell Signaling (Beverly, MA, USA).

### 2.2. Animals and Preparation of Peritoneal Macrophages

C57BL/6 mice (6–8 weeks old, 17–21 g) from Daehan Biolink (Chungbuk, Korea) were maintained under standard care conditions and used for experiments according to the guidelines established by the Institutional Animal Care and Use Committee at Sungkyunkwan University (Suwon, Korea). All animal experiments were carried out by guidelines of the National Institute of Health for the Care and Use of Laboratory Animals (NIH Publication 80–23, revised in 1996) and with approval of the Institutional Animal Care and Use Committee at Sungkyunkwan University (Suwon, Korea). Peritoneal exudates were prepared by intraperitoneal injection with 4% sterile thioglycollate broth (1.0 ml, Difco Laboratories, Detroit, MI) for 4 d, according to previous method [20]. Exudates were washed three times using RPMI 1640 media containing 10% FBS, and peritoneal macrophages were plated in culture plates for each experiment.

### 2.3. Preparation of Pj-ME and Phytochemical Profiling

A 95% methanol extract (Code No. FBM090-039) of the aerial parts of* Protium javanicum *Burm. f. (Pj-ME) was obtained from the Plant Extract Bank of the Plant Diversity Research Center (https://extract.kribb.re.kr/, e-mail: mplantext@kribb.re.kr, Daejeon, Korea). Briefly, the dried aerial parts* Protium javanicum *were pulverized to powder using a mechanical grinder after dried at 60°C for 24 h and then passed through a 60-mesh sieve. The dried powders (100 g) were then extracted with 95% methanol (1 l x 3) for 48 h in the soxhlet apparatus as reported previously [21]. The extracts were filtered and concentrated to vacuum at 40°C under reduced pressure in rotary evaporator and dried in desiccators. The yield of the extract was approximately 12.9%. The crude extract was stored in 4°C to use in the experiment.

Phytochemical profiling of Pj-ME was obtained by high performance liquid chromatography (HPLC) analysis with a system composed of a KNAUER (WellChrom) K-1001 HPLC pump, a K-500 4-channel degasser, and a K-2600 fast scanning spectrophotometer [22]. The elution solvents were buffer A (0.1% trifluoroacetic acid in H_2_O) and buffer B (0.08% trifluoroacetic acid in 95% acetonitrile + 5% H_2_O). The gradient processes were as follows: gradient: 0–30% solvent A (0–15 min), 30% solvent A (15–25min), 30–80% solvent A (25–45 min), and 80% solvent A (45–55 min). The peaks were detected at OD_370_ nm using a Phenomenex Gemini C_18_ ODS (250×4.6 mm, 5 *μ*m). Resveratrol, quercetin, kaempferol, and luteolin were used as reference compounds. The conditions are described in Table 1. Compound analysis was performed by UPLC/HRMS (Orbitrap) analyses using Shimadzu Ultra Performance LCMS 8050 system (Shimadzu, Kyoto, Japan) with a triple quadrupole mass spectrometer equipped with electrospray ionization (ESI) source operating in negative mode (Lab Solutions software version 5.2 (Shimadzu), as reported previously [23, 24]).

### 2.4. Expression Vector Construction

Vectors were constructed by amplification, using standard protocols with competent* E. coli* (DH5*α*). FLAG-MyD88, CFP-TRIF, MyC-Syk, and HA-Src were used as reported. Luciferase constructs that contained NF-*κ*B binding sites were used as previously reported [25, 26]. All constructs were confirmed by DNA sequencing.

### 2.5. Cell Culture and Drug Treatment

The mouse-derived RAW264.7 and HEK293 cells and peritoneal macrophages were cultured in RPMI 1640 medium and Dulbecco's modified Eagle's medium (DMEM), respectively. Both were supplemented with 10% FBS. The cells were grown at 37°C with 5% CO_2_. Pj-ME stock solution (100 *μ*g/ml) was prepared using DMSO.

### 2.6. Nitric Oxide Determination

RAW264.7 cells (1 x 10^6^ cells/ml) were preincubated for 18 h in a CO_2_ incubator, treated with Pj-ME (0-200 *μ*g/ml) or standard compound L-NAME for 30mins, and then incubated with LPS (1 *μ*g/ml) for 24 h. The effect of Pj-ME on NO levels was determined using Griess reagents as described previously [27].

### 2.7. Cell Viability

RAW264.7 cells were incubated in the presence of Pj-ME for 24 h. 10 *μ*l of MTT solution (10 mg/ml in PBS, pH 7.4) was added, and the cells were incubated for 3 h as reported [28, 29]. The reaction was stopped by adding 15% sodium dodecyl sulfate. The samples were then incubated for an additional 24h. The absorbance was calculated at 570 nm based on the control.

### 2.8. RT-PCR Analysis

Total RNA was extracted from RAW264.7 cells incubated with Pj-ME for 30mins and with LPS (1 *μ*g/ml) for 6h using TRIzol reagent (Gibco BRL) according to the manufacturer's instructions. cDNA was prepared using a cDNA synthesis kit (Applied Biosystems #4368814, Foster City, CA, USA) according to the manufacturer's protocol. Semiquantitative RT-PCR gene expression analysis was performed by adding 2 *μ*l of cDNA, 1 *μ*l of forward 5' primer, 1 *μ*l of reverse 3' primer, and 6 *μ*l diethyl pyrocarbonate (DEPC) in 10 *μ*l of PCR premix. Analysis was performed in an RT-thermal cycler (Bio-Rad, Hercules, CA, USA) as reported previously [30, 31]. Primer sequences are listed in Table 2.

### 2.9. Preparation of Whole Cell Lysates and Nuclear Extracts and Western Blot Analysis

For lysis, cultured cells (5 × 10^6^ cells/ml of RAW264.7 and HEK293 cells) washed with cold PBS containing 1 mM sodium orthovanadate were treated with lysis buffer (20 mM Tris-HCl, pH 7.4, 2 mM EGTA, 1 mM sodium orthovanadate, 2 mM EDTA, 1% Triton X-100, 1 mM dithiothreitol, 50 mM *β*-glycerol phosphate, 2 mM PMSF, 10 *μ*g/ml aprotinin, 1 mM benzamide, 10 *μ*g/ml pepstatin, and 10% glycerol,) [32]. The whole cell lysates were prepared with supernatant after centrifugation at 16,000 g for 10 min at 4°C. To prepare membrane fraction, washed cells were lysed in 500 *μ*l lysis buffer and then centrifuged at 19,326 × g for 1 min. The supernatant was further centrifuged at 14,000 rpm for 1 h at 4°C to make membrane and cytosolic fractions as the second step. Finally, the pellet was also treated with extraction buffer (without Triton X-100) [33]. Nuclear lysates were prepared in a three-step procedure. After treatment, cells were harvested, washed with 1 × PBS, and lysed in 500 *μ*l lysis buffer composed of 50 mM KCl, 1 mM PMSF, 100 *μ*M dithiothreitol (DTT) 10 *μ*g/ml leupeptin, 0.5% Nonidet P-40, 20 *μ*g/ml aprotinin, and 25 mM HEPES (pH 7.8) and on ice for 4 min. Cell lysates were then centrifuged at 19,326 × g for 1 min in a microcentrifuge. Secondly, the pellet (the nuclear fraction) was washed in washing buffer (lysis buffer but without Nonidet P-40). Finally, nuclei were prepared by incubation with an extraction buffer (lysis buffer including 10% glycerol and 500 mM KCl). The nuclei/extraction buffer mixture was frozen at -80°C, thawed on ice, and centrifuged at 19,326 × g for 5 min to obtain supernatant part as a nuclear extract. The levels of proteins from whole lysates, membrane fractions, or nuclear extract were analyzed by Western blotting through separating proteins on 10% or 12% SDS-polyacrylamide gels, transferring the proteins to polyvinylidene difluoride (PVDF) membranes, and blocking the membrane in Tris-buffered saline containing 3% bovine serum albumin [34, 35]. Mouse monoclonal antibodies directed against p50, p65, I*κ*B*α*, IKK*α*/*β*, AKT, p85, and *β*-actin (Cell Signaling) were used to detect phosphorylated and total proteins. Following incubation with primary antibodies, blots were washed three times with TBS/Tween 20 before 1 h incubation with secondary anti-mouse or anti-rabbit antibodies. After secondary treatment, blots were again washed with TBS/Tween 20 and then processed for detection using a chemiluminescence system. Proteins were visualized using an ECL system (Amersham, Little Chalfont, Buckinghamshire, UK) as previously reported [36].

### 2.10. Reporter Gene Activity Assay

HEK293 cells were transfected with plasmids expressing NF-*κ*B-luciferase (1 *μ*g/ml), *β*-galactosidase (0.1 *μ*g/ml), and either Flag-MyD88 (1 *μ*g/ml) or HA-Src (1 *μ*g/ml) for 24 h using PEI. Cells were subsequently treated with Pj-ME (0–200 *μ*g/ml) for 24 h. Cells underwent three rounds of freezing and thawing. Cell lysates were used to measure NF-*κ*B-mediated luciferase activity with a luciferase assay system as previously reported [37].

### 2.11. Statistical Analysis

Experiments were conducted independently at a minimum in triplicate. Statistical significance of all data (mean ± standard deviation (SD)) was evaluated by ANOVA/Scheffe's post hoc test and Kruskal-Wallis/Mann-Whitney* U* test using the SPSS program (SPSS Inc., Chicago, IL, USA).

## 3. Results

### 3.1. Pj-ME Suppresses In Vitro Inflammatory Responses

**Figure 1:**
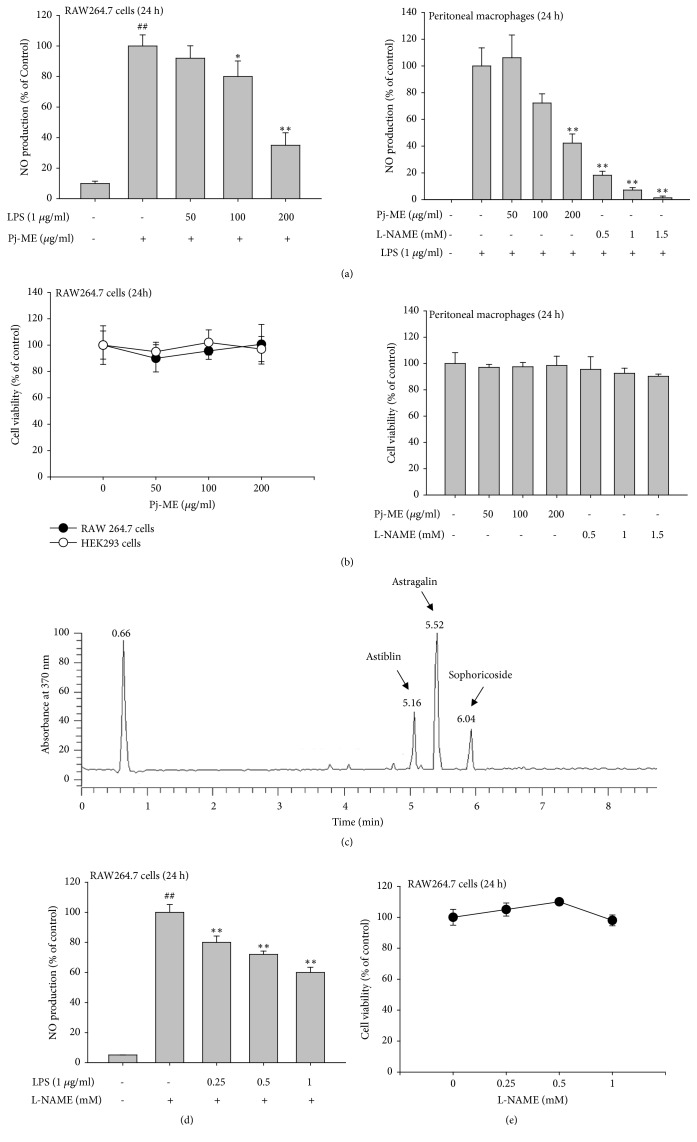
Effects of Pj-ME on NO production in LPS-activated macrophages. ((a) and (d)) Murine macrophage-like RAW264.7 cells or peritoneal macrophages pretreated with Pj-ME (0-200 *μ*g/ml) or L-NAME (0-1 mM) for 30 min and then treated with LPS (1 *μ*g/ml) for 24 h. LPS-induced NO production levels were determined by the Griess assay. ((b) and (e)) To evaluate the cytotoxic activity of Pj-ME or L-NAME, RAW264.7, and HEK293 cells, and peritoneal macrophages were treated with Pj-ME (0-200 *μ*g/ml) and L-NAME (0-1.5 mM) for 24 h. Cell viability was then determined by the MTT assay. (c) Phytochemical fingerprinting was performed by LC/MS spectrophotometric analysis. Putative components were included in each peak. Data ((a), (b), (d), and (e)) expressed as mean ± SD are representative of 3 independent experiments. ^##^: p< 0.01 with respect to the untreated group;  ^*∗*^p< 0.05 and  ^*∗∗*^p< 0.01 with respect to the LPS-treated group.

To assess the potential anti-inflammatory effect of Pj-ME, we used RAW264.7 cells derived from mouse monocytes/macrophages. First, we determined NO production in Pj-ME-treated RAW264.7 cells exposed to the TLR4 ligand LPS (derived from Gram (-) bacteria). Interestingly, NO production in the LPS-stimulated both* RAW264.7 cells (left panel) and peritoneal macrophages (right panel)* was suppressed by Pj-ME (50-200 *μ*g/ml) in a dose-dependent manner (Figure 1(a)). There was a 78% reduction in NO production using 200 *μ*g/ml of Pj-ME in activated RAW264.7 cells (Figure 1(a)). In addition, an escalating dose of Pj-ME (0-200 *μ*g/ml) did not exhibit cytotoxic effects in both RAW264.7 cells (left panel) and peritoneal macrophages (right panel) under normal culture conditions (Figure 1(b)). We also showed that Pj-ME did not affect cell viability in HEK-293 cells at various concentrations (50-200 *μ*g/ml). This suggests that the inhibitory effect of Pj-ME is not due to nonspecific toxic activity. To identify anti-inflammatory components contained in Pj-ME, we used an LC/MS profiling method with four standard anti-inflammatory compounds: resveratrol, quercetin, kaempferol, and luteolin. We were unable to identify these compounds in Pj-ME, but did identify several other compounds, including astilbin (C_21_H_22_O_11_) at 5.16 min, astragalin (C_21_H_20_O_11_) at 5.52 min, and sophoricoside (C_21_H_22_O_10_) at 6.04 min (Figure 1(c)). Because these compounds have been reported as anti-inflammatory suppressors [38–41], the compounds could be the active agents in the extract. Meanwhile, the standard compound L-NAME reduced the release of NO (Figure 1(d)) in a dose-dependent manner as previously reported [42]. Cell viability of L-NAME was >90% at the treatment concentrations (Figure 1(e)).

### 3.2. Pj-ME Suppresses Inflammatory Gene Expression

**Figure 2:**
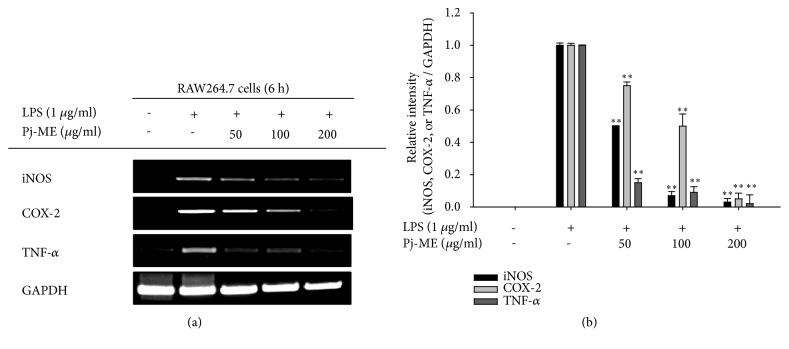
Effect of Pj-ME on inflammatory gene expression. ((a) and (b)) Semiquantitative RT-and real-time PCR analysis was carried out to detect mRNA expression levels of inflammatory genes iNOS, COX-2, and TNF-*α* in RAW264.7 cells pretreated with Pj-ME (50 to 200 *μ*g/ml) for 30 min followed by LPS exposure for 6 h. Data (b) expressed as mean ± SD are representative of 3 independent experiments.  ^*∗∗*^p< 0.01 with respect to the LPS-treated group.

To determine whether Pj-ME-modulated NO production is controlled at the transcriptional or translational level, we chose RAW264.7 cells exposed to LPS and pretreated with escalating doses of Pj-ME, and then we evaluated inflammatory gene levels using both semiquantitative and real-time PCR. We observed that LPS treatment significantly upregulated iNOS, COX-2, and TNF-*α* expression in macrophage-like RAW264.7 cells (Figures 2(a) and 2(b)). Expectedly, both RT-PCR and real-time PCR experiments proved that the upregulation of these genes was suppressed by Pj-ME in a dose-dependent manner (0-200 *μ*g/ml). Notably, Pj-ME (200 *μ*g/ml) suppressed mRNA levels of iNOS, COX-2, and TNF-*α* by more than 70%, according to relative intensity profiling (Figure 2(b)).

### 3.3. Pj-ME Participates in the Regulation of the NF-*κ*B Pathway

**Figure 3:**
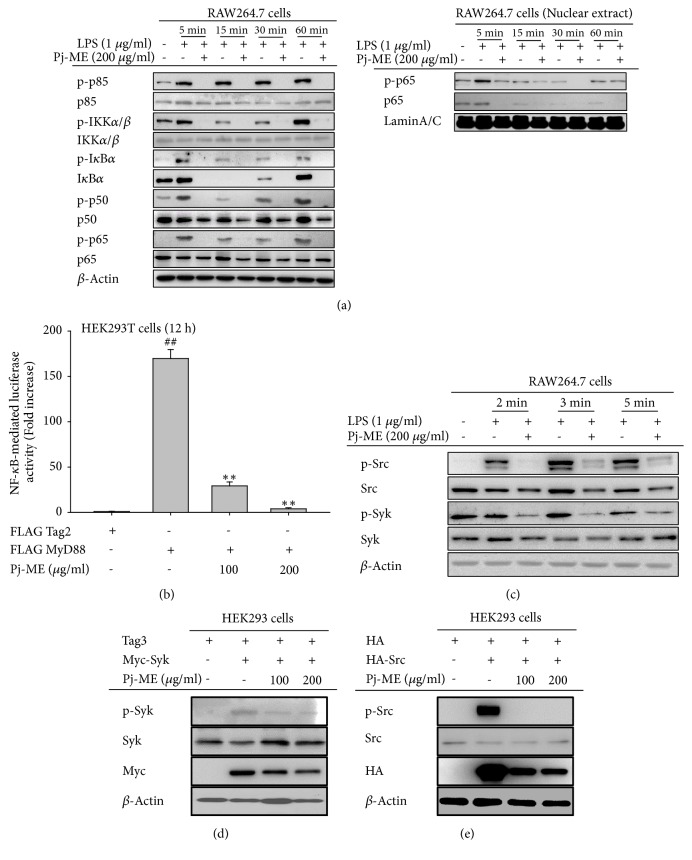
Effect of Pj-ME on the NF-*κ*B and its upstream signaling cascade in LPS-stimulated RAW264.7 cells. ((a) left panel, (a) right panel and (c)) Western blot analysis was performed to detect protein expression levels in whole cell lysates or nuclear extracts from RAW264.7 cells treated with Pj-ME (200 *μ*g/ml) for 30 min followed by LPS exposure (1 *μ*g/ml) over various lengths of incubation times. Levels of phosphorylated and total p85, IKK*α*/*β*, I*κ*B*α*, p50, and p65 at 5, 15, 30, and 60 min, and Syk and Src levels at 2, 3, and 5 min were determined. *β*-Actin was used as a loading control. (b) HEK293 cells cotransfected with NF-*κ*B-Luc (1 *μ*g/ml) and *β*-gal (as transfection control) plasmid constructs were treated with Pj-ME in the presence or absence of the adaptor molecule MyD88 (1 *μ*g/ml). Luciferase activity was measured by using luminescence. ((d) and (e)) Inhibitory activity of Pj-ME (100 and 200 *μ*g/ml) on autophosphorylation of Syk and Src overexpressed in HEK293 cells was determined by Western blot analysis with antibodies specific to phospho-Src or phospho-Syk. Data (b) expressed as mean± SD are representative of 3 independent experiments. ^##^p< 0.01 with respect to untreated group and  ^*∗∗*^p< 0.01 with respect to treated group.

To test the suppressive action of Pj-ME on the intracellular signaling components involved in the activation of NF-*κ*B, we first determined the phosphorylation levels of NF-*κ*B-related signaling molecules (p65/p50 major subunits), which included IKK*α*/*β*, I*κ*B*α*, phospho (p)-p50, and p-p65, using various LPS incubation times (0-60 min) and by immunoblot analysis. We showed that Pj-ME dramatically suppresses the LPS-mediated increase in phosphorylation of p85/PI3K, IKK*α*/*β*, I*κ*B*α*, p50, and p65 after incubation of LPS for 5, 15, 30, and 60 min (Figure 3(a), left panel). Interestingly, nuclear levels of p65 and p-p65 were also reduced by treatment of Pj-ME (200 *μ*g/ml) (Figure 3(a), right panel), demonstrating that upstream regulators of NF-*κ*B are relevant molecular targets of Pj-ME. Since p-p50 and p-p65 are active forms of NF-*κ*B subunits, we also confirmed whether Pj-ME can block upregulated luciferase activity in NF-*κ*B-induced HEK293 cells transfected with MyD88, a major adaptor molecule for NF-*κ*B activation [43]. As shown in Figure 2(b), MyD88 enhanced luciferase activity 175-fold, whereas Pj-ME suppressed luciferase activity by 98% at 200 *μ*g/ml. Interestingly, Pj-ME decreased the phosphorylation of IKK*α*/*β* and I*κ*B*α* at 5 min (Figure 3(a)), which based on our previous results could be mediated by the early activation of the tyrosine kinases Syk and Src [25, 44]. Indeed, phosphorylation of Syk and Src at early time points (2, 3, and 5 mins) was strongly reduced when the cells were treated with 200 *μ*g/ml of Pj-ME (Figure 3(c)). To ensure that these proteins are Pj-ME targets, autophosphorylation levels of Syk or Src were examined by overexpressing the Syk or Src genes in HEK293 cells and using immunoblot analysis as previously reported [45]. Pj-ME suppressed Syk phosphorylation in HEK293 cells in a dose-dependent manner, whereas Src phosphorylation was completely inhibited at 100 and 200 *μ*g/ml (Figures 3(d) and 3(e)). These results suggest that Src or Syk plays an important role in Pj-ME-mediated suppression of inflammatory signaling.

### 3.4. Pj-ME Suppresses STAT3-Induced Inflammation in RAW264.7 Macrophage Cells

**Figure 4:**
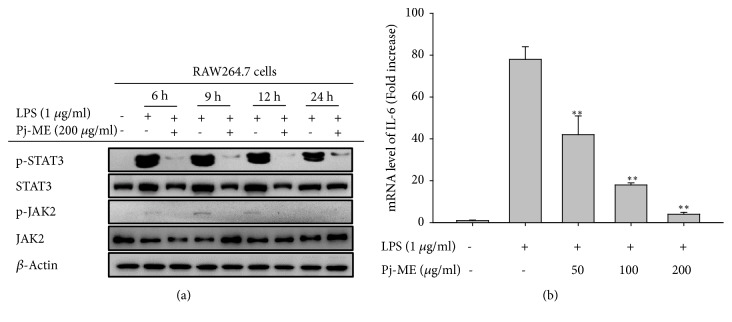
Effect of Pj-ME on the upstream JAK/STAT3 signaling cascade in LPS-stimulated RAW264.7 cells. (a) Western blot analysis was performed to determine protein expression levels in whole cell lysates of RAW264.7 cells treated with Pj-ME (200 *μ*g/ml) for 30 min followed by LPS treatment (1 *μ*g/ml) over different amounts of time. Levels of phosphorylated and total STAT3 and JAK2 at 6, 9, 12, and 24 h were determined with their specific antibodies. *β*-Actin was used as a loading control. (b) STAT3-specific expression of IL-6 was determined by real-time PCR from LPS-treated RAW264.7 cells. Data (b) expressed as mean± SD are representative of 3 independent experiments. ^##^p< 0.01 with respect to untreated group and  ^*∗∗*^p< 0.01 with respect to treated group.

Like NF-*κ*B, STAT3 (signal transducer and activator of transcription 3) is also a major transcription factor that acts in conjunction with NF-*κ*B to induce a number of cytokines and promotes inflammation [46]. It has also been reported that Src tyrosine kinase activates STAT3 and plays a major role in many human tumors [13, 47]. Therefore, we sought to investigate whether Pj-ME suppresses the activation of STAT3 within LPS-stimulated RAW264.7 macrophages. Immunoblot analysis showed an increase in STAT3 phosphorylation and its upstream kinase JAK at later time points (0-24 h) in the LPS-treated cells. However, Pj-ME dramatically decreased LPS-induced phosphorylation of JAK and STAT3 (Figure 4(a)). It is known that IL-6 is major activator of STAT3 [48]. To test whether IL-6 expression is affected by Pj-ME in LPS-treated RAW264.7 cells, we analyzed IL-6 gene expression via quantitative real-time PCR. Notably, Pj-ME inhibited IL-6 expression in a dose-dependent manner (Figure 4(b)).

## 4. Discussion

Since* P. javanicum *has been prescribed as a traditional medicine for treating diarrhea, edema, and leprosy, our aim in this study was to explore the anti-inflammatory mechanism of Pj-ME using* in vitro* experimental conditions [16]. Our results have shown that Pj-ME plays an inhibitory role in NO secretion in LPS-treated RAW264.7 cells, which is secreted as a byproduct involved in inflammation barrier of innate immunity [49]. Moreover, NO production is regulated by* both cancerous and primary* macrophages activated by inflammation-inducing signals at the transcriptional level (Figure 1(a)). Therefore, we investigated whether Pj-ME downregulates inflammatory gene expression in LPS-activated macrophages. As we expected, Pj-ME inhibited the gene expression of iNOS, TNF-*α*, and COX-2 in LPS-stimulated RAW264.7 cells (Figure 2(a)). Moreover, Pj-ME produced these anti-inflammatory effects without affecting cell viability (Figure 1(b)), indicating that the anti-inflammatory effect of Pj-ME is at the transcriptional level and has a specific mode of action not explained by simple cytotoxicity.

Of the many inflammation-regulatory transcription factors, NF-*κ*B and its activating signaling pathway are major regulators of inflammatory gene expression [50]. The possibility that the NF-*κ*B signaling pathway is involved in Pj-ME-regulated anti-inflammatory signaling was explored by inducing NF-*κ*B-driven luciferase activity by MyD88, a major adaptor molecule responsible for NF-*κ*B pathway activation through TLR4 [51, 52]. As expected, Pj-ME (100 and 200 *μ*g/ml) decreased NF-*κ*B-driven luciferase activation in a dose-dependent manner. NF-*κ*B proteins are key regulators of innate and adaptive immune responses, which is triggered by I*κ*B protein degradation followed by I*κ*B kinase (IKK) complex phosphorylation [9]. Based on our previous results, we also investigated NF-*κ*B signaling pathway that regulates transcription factor and tried to identify molecule linking to Pj-ME-mediated anti-inflammatory activity. Using Western blot analysis, we found that the phospho-forms of I*κ*B*α*, IKK*α*/*β*, p50, and p65 were decreased at 5, 15, 30, and 60 mins (Figure 3(a)). The effect of Pj-ME on NF-*κ*B signaling was very strong and motivated us to study the NF-*κ*B upstream signaling in LPS-stimulated RAW264.7 cells. As expected, Pj-ME dramatically suppressed the phosphorylation of Syk and Src, major protein tyrosine kinases known to activate the NF-*κ*B pathway [42, 53], at 2, 3, and 5 mins (Figure 3(b)). These results suggest that Pj-ME targets Syk and Src phosphorylation in the NF-*κ*B signaling pathway. Moreover, there have been several studies that have shown that various natural products with anti-inflammatory activity suppress Src and Syk phosphorylation, supporting the significance of Syk, Src, and NF-*κ*B activation in inflammation and cancer [54–58]. To confirm the molecular target of Pj-ME, we used plasmid constructs to overexpress Syk and Src. Notably, Pj-ME suppressed Syk and Src phosphorylation triggered by their overexpression (Figure 3(c)). Therefore, these results imply that Pj-ME may target tyrosine kinases Syk and Src and inhibit the NF-*κ*B pathway, which would explain the anti-inflammatory activity of this extract.

Although the potential of Pj-ME for modulating gene expression was not fully evaluated, it is arguable from our study that changes in transcription and translation due to the extracts and compounds contribute to the biological effects. Our study also showed that Pj-ME suppresses STAT3 signaling and genes regulated by STAT3, namely IL-6 (Figure 4(b)). However, it is possible that suppression of IL-6 gene expression could be mediated by NF-*κ*B. Moreover, natural compounds like resveratrol have also been reported as inhibitors of inflammation, which includes NO production triggered by STAT3/IL-6 in the tumor microenvironment [59, 60]. Therefore, our findings suggest that the potential of Pj-ME to modulate inflammatory responses could be driven by suppression of inflammatory signaling pathways linked to the activation of NF-*κ*B and STAT3.

By HPLC/MS spectrometry, we have identified several compounds such as astragalin and sophoricoside from Pj-ME. So far, we have not tested whether these compounds were involved in the anti-inflammatory activity of Pj-ME. However, literatures have apparently mentioned that astilbin, astragalin, and sophoricoside are able to suppress the production of inflammatory mediators in macrophages [38–41]. Therefore, it is assumed that the compounds could be the active agents in the extract. The fact that standard anti-inflammatory compounds including resveratrol, quercetin, kaempferol, and luteolin were not detected in this extract (data not shown) also indicates that these flavonoids are not included in Pj-ME. Further detailed study on identification of active components in this extract will be followed by activity-guided fractionation strategy.

**Figure 5:**
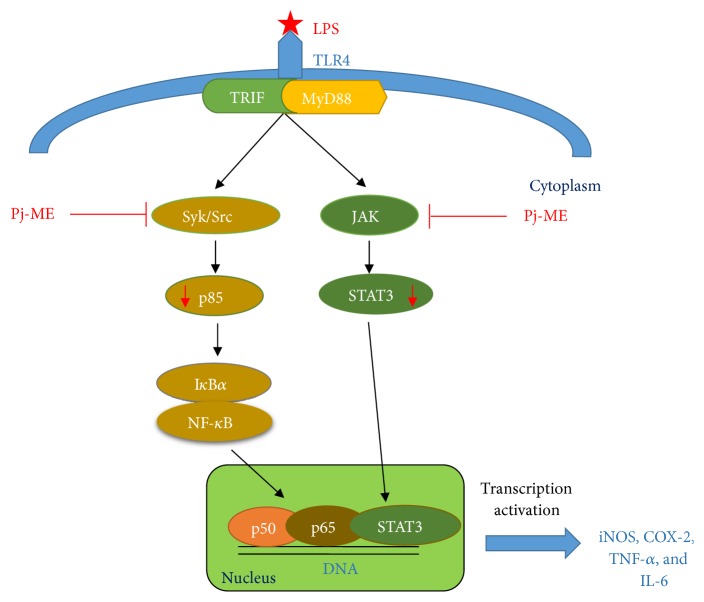
Putative suppressive pathway of Pj-ME in displaying its anti-inflammatory response. It is considered that Pj-ME targets the activation of protein tyrosine kinases such as JAK and Src and Syk linked to the activation of intracellular signaling pathway for the nuclear translocation of NF-*κ*Band STAT3. Suppression of this pathway leads to the downregulation of iNOS-mediated NO production and the expression of other cytokines such as NO and IL-6.

In summary* Protium javanicum *Burm. f. methanol extracts attenuated NF-*κ*B-mediated inflammatory signaling by downregulating Syk and Src phosphorylation in LPS-treated RAW264.7 macrophages. Moreover, Pj-ME inhibited the STAT3 signaling pathway in late time phase, which implies that Src and Syk suppression may play an important role in inhibiting cytokines, like IL-6, and activate STAT3 (Figure 5). Although Pj-ME has been used as an ethnopharmacological remedy, we have provided evidence to support its anti-inflammatory activity, enhancing our understanding of the role of Pj-ME in inflammation. Furthermore, additional preclinical studies using* in vivo* models will be used to establish potential therapeutic uses.

## Figures and Tables

**Table 1:** High performance liquid chromatography (HPLC) condition conditions to analyze active ingredients.

Instrument	KNAUER crop. HPLC system
Column	Phenomenex, Gemini 5 *μ*m C18 110A, 250 X 4.60 mm
Detector	UV/VIS detector (370 nm)
Solvent A	0.1% TFA in H_2_O
Solvent B	0.008% TFA in 95% MeCN + 5% H_2_O
Standard	Dilution with DMSO
Sample treatment	50mg/ml dilution with DMSO
Injection volume	20 *μ*l
Flow rate	1.0 ml/min

**Table 2:** Semiquantitative PCR and real-time PCR primer sequences used in the study.

Primer Name	Direction	Sequence (5' to 3')
	*Semi quantitative PCR*	
iNOS	Forward	CCCTTCCGAAGTTTCTGGCAGCAG
	Reverse	GGCTGTCAGAGCCTCGTGGCTTTGG
COX-2	Forward	CACTACATCCTGACCCACTT
	Reverse	ATGCTCCTGCTTGAGTATGT
TNF-*α*	Forward	TTGACCTCAGCGCTGAGTTG
	Reverse	CCTGTAGCCCACGTCGTAGC
GAPDH	Forward	CACTCACGGCAAATTCAACGGCA
	Reverse	GACTCCACGACATACTCAGCAC

	*Real time PCR*	
IL-6	Forward	CTAGGTTTGCCGAGTAGATCTC
	Reverse	GACAAAGCCAGAGTCCTTCAGAGA

## Data Availability

The data used to support the findings of this study are available from the corresponding author upon request.

## References

[1] Hirahara K., Poholek A., Vahedi G. (2013). Mechanisms underlying helper T-cell plasticity: implications for immune-mediated disease. *The Journal of Allergy and Clinical Immunology*.

[2] Scrivo R., Vasile M., Bartosiewicz I., Valesini G. (2011). Inflammation as ‘common soil’ of the multifactorial diseases. *Autoimmunity Reviews*.

[3] Valledor A. F., Comalada M., Santamaría-Babi L. F., Lloberas J., Celada A. (2010). Macrophage proinflammatory activation and deactivation: a question of balance. *Advances in Immunology*.

[4] De Geus E. D., Vervelde L. (2013). Regulation of macrophage and dendritic cell function by pathogens and through immunomodulation in the avian mucosa. *Developmental & Comparative Immunology*.

[5] Xue J., Sharma V., Hsieh M. H. (2015). Alternatively activated macrophages promote pancreatic fibrosis in chronic pancreatitis. *Nature Communications*.

[6] Libby P. (2007). Inflammatory mechanisms: the molecular basis of inflammation and disease. *Nutrition Reviews*.

[7] Karachaliou N., Gonzalez-Cao M., Crespo G. (2018). Interferon gamma, an important marker of response to immune checkpoint blockade in non-small cell lung cancer and melanoma patients. *Therapeutic Advances in Medical Oncology*.

[8] Bonizzi G., Karin M. (2004). The two NF-kappaB activation pathways and their role in innate and adaptive immunity. *Trends in Immunology*.

[9] Karin M., Greten F. R. (2005). NF-*κ*B: linking inflammation and immunity to cancer development and progression. *Nature Reviews Immunology*.

[10] Karin M., Ben-Neriah Y. (2000). Phosphorylation meets ubiquitination: the control of NF-*κ*B activity. *Annual Review of Immunology*.

[11] Sehgal P. B. (2008). Paradigm shifts in the cell biology of STAT signaling. *Seminars in Cell & Developmental Biology*.

[12] Johnston P. A., Grandis J. R. (2011). STAT3 signaling: anticancer strategies and challenges. *Molecular Interventions*.

[13] Yu C.-L., Meyer D. J., Campbell G. S. (1995). Enhanced DNA-binding activity of a stat3-related protein in cells transformed by the Src oncoprotein. *Science*.

[14] Yue P., Turkson J. (2009). Targeting STAT3 in cancer: How successful are we?. *Expert Opinion on Investigational Drugs*.

[15] Adfa M., Hattori Y., Ninomiya M., Funahashi Y., Yoshimura T., Koketsu M. (2013). Chemical constituents of Indonesian plant Protium javanicum Burm. f. and their antifeedant activities against Coptotermes formosanus Shiraki. *Natural Product Research (Formerly Natural Product Letters)*.

[16] Adfa M., Yoshimura T., Komura K., Koketsu M. (2010). Antitermite activities of coumarin derivatives and scopoletin from Protium javanicum Burm. f. *Journal of Chemical Ecology*.

[17] Ahuja A., Kim J. H., Kim J.-H., Yi Y.-S., Cho J. Y. (2018). Functional role of ginseng-derived compounds in cancer. *Journal of Ginseng Research*.

[18] Aziz N., Kim M.-Y., Cho J. Y. (2018). Anti-inflammatory effects of luteolin: A review of in vitro, in vivo, and in silico studies. *Journal of Ethnopharmacology*.

[19] Kim J. H., Yi Y.-S., Kim M.-Y., Cho J. Y. (2017). Role of ginsenosides, the main active components of Panax ginseng, in inflammatory responses and diseases. *Journal of Ginseng Research*.

[20] Kim H. G., Kim M. Y., Cho J. Y. (2018). Alisma canaliculatum ethanol extract suppresses inflammatory responses in LPS-stimulated macrophages, HCl/EtOH-induced gastritis, and DSS-triggered colitis by targeting Src/Syk and TAK1 activities. *Journal of Ethnopharmacology*.

[21] Jeong S., Kim S., Kim H. G. (2019). Mycetia cauliflora methanol extract exerts anti-inflammatory activity by directly targeting PDK1 in the NF-*κ*B pathway. *Journal of Ethnopharmacology*.

[22] Wang H.-P., Zhang Y.-B., Yang X.-W., Zhao D.-Q., Wang Y.-P. (2016). Rapid characterization of ginsenosides in the roots and rhizomes of panax ginseng by UPLC-DAD-QTOF-MS/MS and simultaneous determination of 19 ginsenosides by HPLC-ESI-MS. *Journal of Ginseng Research*.

[23] Sun Z., Zuo L., Sun T. (2017). Chemical profiling and quantification of XueBiJing injection, a systematic quality control strategy using UHPLC-Q Exactive hybrid quadrupole-orbitrap high-resolution mass spectrometry. *Scientific Reports*.

[24] Pavlović I., Petřík I., Tarkowská D. (2018). Correlations between phytohormones and drought tolerance in selected brassica crops: chinese cabbage, white cabbage and kale. *International Journal of Molecular Sciences*.

[25] Kim H. G., Choi S., Lee J. (2018). Src Is a prime target inhibited by Celtis choseniana methanol extract in its anti-inflammatory action. *Evidence-based Complementary and Alternative Medicine*.

[26] Hunto S. T., Shin K. K., Kim H. G. (2018). Phosphatidylinositide 3-kinase contributes to the anti-inflammatory effect of *Abutilon crispum* L. Medik methanol extract. *Evidence-Based Complementary and Alternative Medicine*.

[27] Han S. Y., Kim J., Kim E. (2018). AKT-targeted anti-inflammatory activity of Panax ginseng calyx ethanolic extract. *Journal of Ginseng Research*.

[28] Pauwels R., Balzarini J., Baba M. (1988). Rapid and automated tetrazolium-based colorimetric assay for the detection of anti-HIV compounds. *Journal of Virological Methods*.

[29] Yayeh T., Jung K.-H., Jeong H. Y. (2012). Korean Red Ginseng saponin fraction downregulates proinflammatory mediators in LPS stimulated RAW264.7 cells and protects mice against endotoxic shock. *Journal of Ginseng Research*.

[30] Lee Y. G., Chain B. M., Cho J. Y. (2009). Distinct role of spleen tyrosine kinase in the early phosphorylation of inhibitor of kappaB alpha via activation of the phosphoinositide-3-kinase and Akt pathways. *The International Journal of Biochemistry & Cell Biology*.

[31] Zhang R., Zhu J., Cao H.-Z. (2013). Isolation and characterization of LHT-type plant amino acid transporter gene from Panax ginseng Meyer. *Journal of Ginseng Research*.

[32] Kim H. D., Ha S. E., Kang J. R., Park J. K. (2010). Effect of Korean red ginseng extract on cell death responses in peroxynitrite-treated keratinocytes. *Journal of Ginseng Research*.

[33] Lin W.-J., Gary J. D., Yang M. C., Clarke S., Herschman H. R. (1996). The mammalian immediate-early TIS21 protein and the leukemia-associated BTG1 protein interact with a protein-arginine N-methyltransferase. *The Journal of Biological Chemistry*.

[34] Kim E.-H., Lee M.-J., Kim I.-H., Pyo S., Choi K.-T., Rhee D.-K. (2010). Anti-apoptotic effects of red ginseng on oxidative stress induced by hydrogen peroxide in SK-N-SH cells. *Journal of Ginseng Research*.

[35] Song K. C., Chang T.-S., Lee H., Kim J., Park J. H., Hwang G. S. (2012). Processed *Panax ginseng*, sun ginseng increases type I collagen by regulating MMP-1 and TIMP-1 expression in human dermal fibroblasts. *Journal of Ginseng Research*.

[36] Lee J.-A., Lee M.-Y., Shin I.-S., Seo C.-S., Ha H., Shin H. K. (2012). Anti-inflammatory effects of amomum compactum on RAW 264.7 cells via induction of heme oxygenase-1. *Archives of Pharmacal Research*.

[37] Yang W. S., Kim D., Yi Y.-S. (2017). AKT-targeted anti-inflammatory activity of the methanol extract of Chrysanthemum indicum var. albescens. *Journal of Ethnopharmacology*.

[38] Di T.-T., Ruan Z.-T., Zhao J.-X. (2016). Astilbin inhibits Th17 cell differentiation and ameliorates imiquimod-induced psoriasis-like skin lesions in BALB/c mice via Jak3/Stat3 signaling pathway. *International Immunopharmacology*.

[39] Wang S.-W., Xu Y., Weng Y.-Y. (2018). Astilbin ameliorates cisplatin-induced nephrotoxicity through reducing oxidative stress and inflammation. *Food and Chemical Toxicology*.

[40] Ma Z., Piao T., Wang Y., Liu J. (2015). Astragalin inhibits IL-1*β*-induced inflammatory mediators production in human osteoarthritis chondrocyte by inhibiting NF-*κ*B and MAPK activation. *International Immunopharmacology*.

[41] Li W., Lu Y. (2018). Hepatoprotective effects of sophoricoside against fructose-induced liver injury via regulating lipid metabolism, oxidation, and inflammation in mice. *Journal of Food Science*.

[42] Kim Y. J., Deok J., Kim S. (2017). Anti-inflammatory effect of Piper attenuatum methanol extract in LPS-stimulated inflammatory responses. *Evidence-based Complementary and Alternative Medicine*.

[43] Park J. G., Son Y. J., Yoo B. C. (2017). Syk plays a critical role in the expression and activation of IRAK1 in LPS-treated macrophages. *Mediators of Inflammation*.

[44] Yu J. S., Kim J. H., Lee S., Jung K., Kim K. H., Cho J. Y. (2017). rc/Syk-targeted anti-inflammatory actions of triterpenoidal saponins from Gac (*Momordica cochinchinensis*) seeds. *American Journal of Chinese Medicine*.

[45] Yoon J. Y., Jeong H. Y., Kim S. H. (2013). Methanol extract of *Evodia lepta* displays Syk/Src-targeted anti-inflammatory activity. *Journal of Ethnopharmacology*.

[46] Yang J., Liao X., Agarwal M. K., Barnes L., Auron P. E., Stark G. R. (2007). Unphosphorylated STAT3 accumulates in response to IL-6 and activates transcription by binding to NF*κ*B. *Genes & Development*.

[47] Garcia R., Bowman T. L., Niu G. (2001). Constitutive activation of Stat3 by the Src and JAK tyrosine kinases participates in growth regulation of human breast carcinoma cells. *Oncogene*.

[48] Naugler W. E., Sakurai T., Kim S. (2007). Gender disparity in liver cancer due to sex differences in MyD88-dependent IL-6 production. *Science*.

[49] Brennan P. A., Downie I. P., Langdon J. D. (1999). Emerging role of nitric oxide in cancer. *The British Journal of Oral & Maxillofacial Surgery*.

[50] Kim E., Kang Y.-G., Kim J. H. (2018). The antioxidant and anti-inflammatory activities of 8-hydroxydaidzein (8-HD) in activated macrophage-like RAW264.7 cells. *International Journal of Molecular Sciences*.

[51] Jain A., Kaczanowska S., Davila E. (2014). IL-1 receptor-associated kinase signaling and its role in inflammation, cancer progression, and therapy resistance. *Frontiers in Immunology*.

[52] Schmitz F., Mages J., Heit A., Lang R., Wagner H. (2004). Transcriptional activation induced in macrophages by Toll-like receptor (TLR) ligands: from expression profiling to a model of TLR signaling. *European Journal of Immunology*.

[53] Yoo S., Kim M.-Y., Cho J. Y. (2018). Syk and Src-targeted anti-inflammatory activity of aripiprazole, an atypical antipsychotic. *Biochemical Pharmacology*.

[54] Park J. G., Kim S. C., Kim Y. H. (2016). Anti-inflammatory and antinociceptive activities of anthraquinone-2-carboxylic acid. *Mediators of Inflammation*.

[55] Sung N. Y., Kim M.-Y., Cho J. Y. (2015). Scutellarein reduces inflammatory responses by inhibiting Src kinase activity. *Korean Journal of Physiology & Pharmacology*.

[56] Kim S. H., Park J. G., Lee J. (2015). The dietary flavonoid Kaempferol mediates anti-inflammatory responses via the Src, Syk, IRAK1, and IRAK4 molecular targets. *Mediators of Inflammation*.

[57] Arias-Salgado E. G., Lizano S., Sarkar S., Brugge J. S., Ginsberg M. H., Shattil S. J. (2003). Src kinase activation by direct interaction with the integrin *β* cytoplasmic domain. *Proceedings of the National Acadamy of Sciences of the United States of America*.

[58] Rowley R. B., Burkhardt A. L., Chao H.-G., Matsueda G. R., Bolen J. B. (1995). Syk protein-tyrosine kinase is regulated by tyrosine-phosphorylated Ig*α*/Ig*β* immunoreceptor tyrosine activation motif binding and autophosphorylation. *The Journal of Biological Chemistry*.

[59] Burdelya L., Kujawski M., Niu G. (2005). Stat3 activity in melanoma cells affects migration of immune effector cells and nitric oxide-mediated antitumor effects. *The Journal of Immunology*.

[60] Kotha A., Sekharam M., Cilenti L. (2006). Resveratrol inhibits Src and Stat3 signaling and induces the apoptosis of malignant cells containing activated Stat3 protein. *Molecular Cancer Therapeutics*.

